# Efficient Tissue Culture Method Based on Clustered Bud Proliferation for Producing High-Quality *Arundo donax* Seedlings

**DOI:** 10.3390/plants14192978

**Published:** 2025-09-25

**Authors:** Jialin Guo, Mingchen Yu, Wei Li, Gangqiang Cao, Luyue Zhang, Weiwei Chen, Zhengqing Xie, Gongyao Shi, Fang Wei, Baoming Tian

**Affiliations:** Henan International Joint Laboratory of Crop Gene Resources and Improvements, School of Agricultural Sciences, Zhengzhou University, Zhengzhou 450001, China; jialinguo1990@163.com (J.G.); yumingchen0619@gs.zzu.edu.cn (M.Y.); weili21@gs.zzu.edu.cn (W.L.); caogq@zzu.edu.cn (G.C.); luyuezhang@zzu.edu.cn (L.Z.); weiwei_chen15134@zzu.edu.cn (W.C.); zqxie@zzu.edu.cn (Z.X.); shigy@zzu.edu.cn (G.S.)

**Keywords:** clustered shoots, giant reed, growth regulator, plant hormone, reproduction

## Abstract

For its rapid growth, high yield, and broad adaptability, *Arundo donax* is widely used in various applications, yielding considerable economic and ecological benefits. However, widespread cultivation is challenging because *A. donax* can only be propagated asexually. In this study, a tissue culture method was developed using the clustered bud proliferation pathway. The explant type, disinfection method, induction medium, proliferation medium, and rooting medium were optimized to efficiently harvest high-quality *A. donax* seedlings. Using axillary buds with whole cane fragments as the most suitable explants, they were first sterilized with 75% alcohol for 30 s and then disinfected with 0.1% mercuric chloride for 5 min. 97.8% of explants could successfully form clustered buds on Murashige and Skoog (MS) medium supplemented with 5.0 mg/L 6-benzylaminopurine (6-BA) and 1.0 mg/L 3-indolebutyric acid (IBA). Each individual bud achieved efficient propagation with a proliferation coefficient as high as 33.3 on MS medium supplemented with 4.0 mg/L 6-BA and 1.0 mg/L IBA. In addition, all buds were capable of rooting on 1/2 MS medium supplemented with 0.5 mg/L 1-naphthaleneacetic acid (NAA). The resultant rooted seedlings survived and developed into plantlets, averaging 44.84 cm in height and 2.54 mm in thickness, following a 30-day acclimation period. This protocol provides a robust foundation for the large-scale, high-quality propagation of *A. donax*, supporting its broader application in ecological restoration and bioresource industries.

## 1. Introduction

*Arundo donax*, commonly known as the giant reed, is a perennial herbaceous plant belonging to the Poaceae family. It originates from Asia but is now invasive in numerous global regions [1,2]. With the thick and upright canes and flat and broad leaves, the morphology of *A. donax* closely resembles that of reeds [3] and bamboo [4]; however, they are classified under different genera. *A. donax* exhibits rapid growth, resulting in a high biomass yield [5,6,7]. Its dry mass can exceed 30 t per hectare in a single year, even in the absence of water and nutritional restrictions [8,9]. Under specific cultivation conditions, the dry weight of *A. donax* may exceed 100 t per hectare [10].

In addition to its rapid growth rate and high biomass yield, *A. donax* is characterized by its tolerance to adverse environmental conditions [11,12,13] and capacity to thrive in various soil types [14,15,16] and climatic conditions [17,18,19]. These characteristics enable *A. donax* to well grow on marginal land, thus avoiding the completion with grain crops for cultivated land. Recent studies have indicated that *A. donax* possesses considerable economic value and is widely used in various applications, including vibrating plates for woodwind instruments [20,21], edible and medicinal fungal cultivation [22,23,24], forage production [25,26], fiber extraction [27,28], biomass energy production [29,30], and ecological protection and restoration [31,32,33,34]. Correspondingly, the demand for *A. donax* seedlings has increased; however, scaling up production poses challenges due to the sterility and reliance of the species on asexual propagation [35,36].

Traditionally, *A. donax* is mainly propagated by root-splitting and stem-cutting methods. Rooting-splitting propagation involves planting rhizomes after splitting them into clusters containing 4–5 buds. This method exhibits a straightforward operation and high survival rate; however, it has a poor proliferation coefficient, is costly and labor-intensive, and results in the loss of the mother plant [37,38,39]. Stem-cutting propagation involves directly inserting stem segments into the soil. This method is simple to implement and exhibits a higher proliferation coefficient than that observed for rooting-splitting. However, stem segments can only be inserted into an open field during specific months because the development of axillary buds requires appropriate temperature and light conditions. Furthermore, planting stem segments in unsuitable seasons leads to rooting difficulties and a decreased survival rate [40,41,42]. Although the rooting and survival rates can be improved via hydroponic culture for some time before the stem segments are transplanted into the field, the stem segments should be harvested in specific months [43]. Currently, the plant is traditionally propagated involving rhizomes and stem nodes; however, the aforementioned limitations prevent the application of these two methods in the industrial production of *A. donax* seedlings. Therefore, more efficient propagation approaches in terms of both the number of *A. donax* seedlings obtained and time required to propagate them are required.

Recent advancements in scientific research have established tissue culture propagation as an effective method for producing a substantial quantity of morphologically, physiologically, and genetically identical clonal plants within a short timeframe, irrespective of seasonal and other factors. Currently, certain researchers have explored the application of tissue culture methods for producing *A. donax* seedlings; however, different genotypes of *A. donax* are compatible with distinct methods [44,45,46]. Antal et al. [44] and Cavallaro et al. [45] applied a tissue culture method based on clustered bud proliferation. This method utilizes the regenerative capacity of axillary buds, with explants initially induced to form clustered buds, which are subsequently used to yield seedlings via subculture and rooting processes. Ozudogru et al. [46] and Valli et al. [47] utilized a tissue culture method based on callus induction. This method utilizes the totipotency of plant cells, where explants are first induced to form calli, which are then used to yield seedlings through callus proliferation, differentiation, and root formation. In comparison, the clustered bud proliferation method is simpler and requires less time and fewer operational requirements. However, its effectiveness is influenced by various factors, including explant types, disinfection methods, culture media types, hormone types and ratios, and culture conditions [44,45,48,49,50]. At present, the proliferation coefficients of reported methods ranged from 5.6 to 16 [44,45,49], which should be further improved to meet the increasing demand for *A. donax* seedlings.

In this study, we hypothesize that a tissue culture method based on clustered bud proliferation with a high proliferation coefficient for *A. donax* propagation will be developed by optimizing the explant type, disinfection method, induction medium, proliferation medium, and rooting medium. The findings of this study are anticipated to advance our understanding of efficient clonal propagation strategies for sterile perennial grasses and provide a theoretical basis for the large-scale, standardized production of high-quality *A. donax* seedlings through tissue culture.

## 2. Results

### 2.1. Effects of Various Explants on Bud Induction

**Figure 1:**
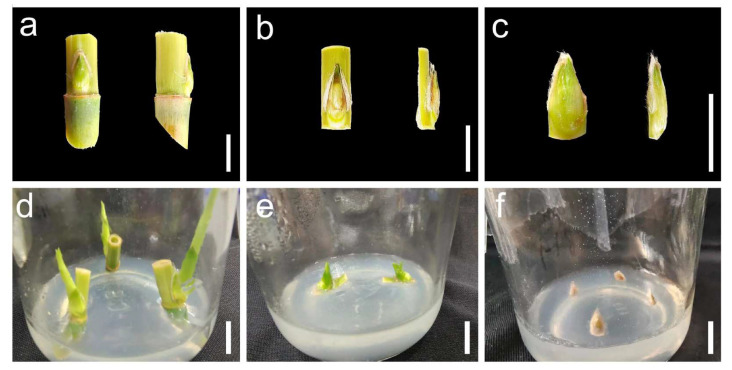
Effects of different explants on bud induction. (**a**–**c**) Morphological characteristics of axillary buds with whole cane fragments, axillary buds with incomplete cane fragments, and axillary buds without cane fragments, respectively; (**d**–**f**) Induction effects of axillary buds with whole cane fragments, axillary buds with incomplete cane fragments, and axillary buds without cane fragments cultured on MS medium supplemented with 5.0 mg/L 6-BA and 1.0 mg/L IBA for 15 days, respectively. Bars = 1 cm.

When axillary buds with whole cane fragments were used as explants, the axillary buds sprouted more easily, and the induced shoots were longer after a 15-day culture on Murashige and Skoog (MS, Beijing Solarbio Science & Technology Co., Ltd., Beijing, China) medium supplemented with 5.0 mg/L 6-benzylaminopurine (6-BA, Beijing Solarbio Science & Technology Co., Ltd., Beijing, China) and 1.0 mg/L 3-indolebutyric acid (IBA, Beijing Solarbio Science & Technology Co., Ltd., Beijing, China) (Figure 1). Upon longitudinally cutting the cane fragment, the axillary buds with incomplete cane fragments successfully sprouted; nevertheless, the length of the induced shoots was shorter than that of axillary buds with whole cane fragments. However, if the axillary buds were individually cut off, the explants of axillary buds without cane fragments failed to initiate sprouting, and the browning phenomenon was severe. Therefore, explants of axillary buds with whole cane fragments were selected as the optimal explants for inducing clustered buds.

### 2.2. Effects of Various Disinfectants on Explant Disinfection

To identify the most suitable disinfectant, the explants were first treated with 75% alcohol for 30 s and subsequently treated with sodium hypochlorite or mercury chloride (Shanghai Hushi Laboratory Equipment Co., Ltd., Shanghai, China) solution. Following a 7-day culture on MS medium supplemented with 5.0 mg/L 6-BA and 1.0 mg/L IBA, the contamination and mortality rates of the explants were investigated (Table 1). Upon treatment of the explants with 2% sodium hypochlorite for 10 min, all explants survived; however, the contamination rate reached 74.4%. When the sodium hypochlorite concentration increased to 10%, the contamination rate of the explants decreased significantly to 52.2%; however, the mortality rate increased to 58.9%. When the explants were treated with 0.1% mercury chloride for 3 min, all explants survived, with a contamination rate of 35.6%. When the treatment time with 0.1% mercury chloride increased to 5 min, the contamination rate of the explants significantly decreased to 17.8%, while the mortality rate increased to 22.2%. Comparing these six different treatments revealed that explants treated with 0.1% mercury chloride for 5 min statistically exhibited the lowest contamination rate and a relatively low mortality rate. Therefore, this treatment method was the final established method for explant disinfection.

### 2.3. Determination of Induction Medium

Sterile explants were cultured on MS media supplemented with various combinations of cytokinins and auxins to induce bud germination. The induction effects were determined by investigating the induction and browning rates of explants after culturing for 30 days. As shown in Table 2, the combination of 5.0 mg/L 6-BA and 1.0 mg/L 2,4-dichlorophenoxyacetic acid (2,4-D, Beijing Solarbio Science & Technology Co., Ltd., Beijing, China) resulted in a low sprouting rate among the explants, with an induction rate of merely 3.3%, while the browning rate reached 25.6%. Maintaining the cytokinin at 5.0 mg/L 6-BA while substituting 2,4-D with 1-naphthaleneacetic acid (NAA, Beijing Solarbio Science & Technology Co., Ltd., Beijing, China) resulted in a significant increase in the induction rate to 72.2% and significant decrease in the browning rate to 4.4%. Furthermore, substituting 2,4-D with IBA resulted in a significant increase in the induction rate to 97.8% and significant decrease in the browning rate to a minimal value of 1.1%.

To explore the potential for improved induction effects from other cytokinins, the auxin was maintained at 1.0 mg/L IBA, while the cytokinin of 5.0 mg/L 6-BA was substituted with kinetin (KT) or thidiazuron (TDZ). The results showed that the induction rates of the explants treated with KT and TDZ significantly decreased to 87.8% and 81.1%, respectively. The browning rates increased to 5.6% and 4.4%, respectively, which were not significantly different from those of 6-BA. The combination of 5.0 mg/L 6-BA and 1.0 mg/L IBA exhibited the most effective induction among the five different treatments analyzed. Therefore, the induction medium was identified as MS medium supplemented with 5.0 mg/L 6-BA and 1.0 mg/L IBA.

### 2.4. Determination of Proliferation Medium

**Figure 2:**
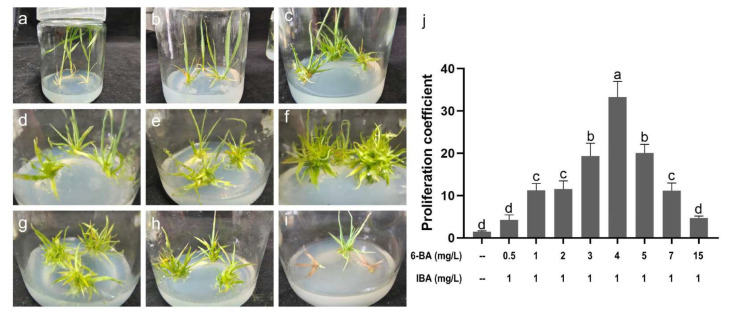
Effects of various combinations of 6-BA and IBA on bud proliferation. (**a**) Proliferation effect of individual buds cultured on MS medium not supplemented with hormones for 30 days; (**b**–**i**) Proliferation effect of individual buds cultured on MS medium containing 0.5, 1.0, 2.0, 3.0, 4.0, 5.0, 7.0, and 15.0 mg/L 6-BA and 1.0 mg/L IBA for 30 days, respectively; (**j**) Proliferation coefficient of individual buds cultured on various media for 30 days; the means (*n* = 3) denoted by the same letter are not significantly different according to the LSD test at *p* = 0.05.

The clustered buds induced on MS + 5.0 mg/L 6-BA + 1.0 mg/L IBA were segmented into individual buds and transferred to MS medium supplemented with various combinations of 6-BA and IBA for bud proliferation. After culturing for 30 days, the growth conditions and proliferation coefficients were assessed (Figure 2). In the absence of hormones in the MS medium, the cultured individual buds were mainly elongated, with minimal proliferation activity, resulting in a proliferation coefficient of only 1.5. The addition of 0.5 mg/L 6-BA and 1.0 mg/L IBA to the MS medium did not result in a significant proliferation phenomenon, with a proliferation coefficient of cultured buds measured at 4.3. This was similar to that of medium not supplemented with hormones. Further, when the auxin was maintained at 1.0 mg/L IBA, an increase in the concentration of cytokinin 6-BA from 1.0 mg/L to 7.0 mg/L resulted in enhanced proliferation activity, with the proliferation coefficient significantly increasing to a range of 11.1–33.3. In addition, the proliferation coefficient exhibited a trend of first increasing and then decreasing, with the maximum proliferation coefficient occurring at 4.0 mg/L 6-BA. This was significantly higher than those in other treatment groups. However, when the concentration of 6-BA was increased to 15.0 mg/L, the growth condition of the single bud cultured was similar to that of the 0.5 mg/L treatment, with an insignificant difference in the proliferation coefficient of 4.7. Consequently, an MS medium supplemented with 4.0 mg/L 6-BA and 1.0 mg/L IBA was optimal for bud proliferation.

### 2.5. Determination of the Rooting Medium

**Figure 3:**
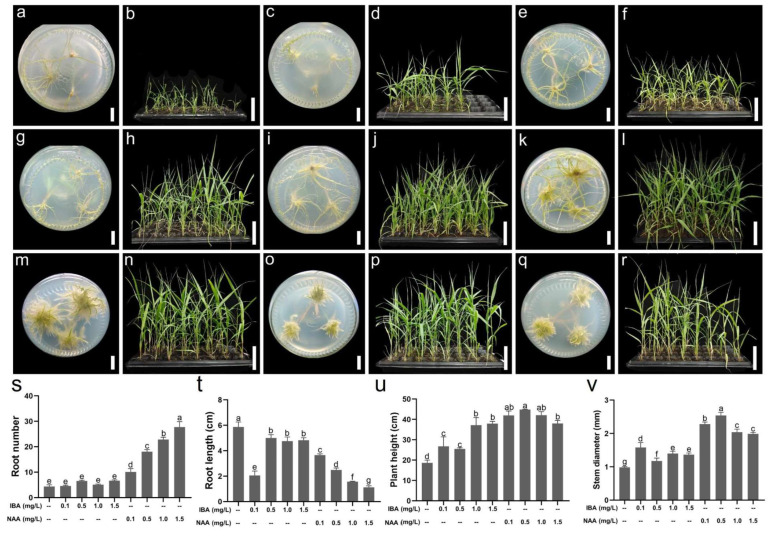
Rooting and acclimation effects of seedlings derived from various media. (**a**,**b**) Rooting and acclimation effects of seedlings from 1/2 MS containing no hormones; (**c**,**e**,**g**,**i**) Rooting effects of seedlings from 1/2 MS containing 0.1, 0.5, 1.0, 1.5 mg/L IBA, respectively; (**d**,**f**,**h**,**j**) Acclimation effects of seedlings from 1/2 MS containing 0.1, 0.5, 1.0, 1.5 mg/L IBA, respectively; (**k**,**m**,**o**,**q**) Rooting effects of seedlings from 1/2 MS containing 0.1, 0.5, 1.0, 1.5 mg/L NAA, respectively; (**l**,**n**,**p**,**r**) Acclimation effects of seedlings from 1/2 MS containing 0.1, 0.5, 1.0, 1.5 mg/L NAA, respectively; (**s**,**t**) Root number and root length of seedlings from various media; (**u**,**v**) Plant height and stem diameter of seedlings from various media following acclimation for 30 days. Bars = 1 cm in (**a**,**c**,**e**,**g**,**i**,**k**,**m**,**o**,**q**), 10 cm in (**b**,**d**,**f**,**h**,**j**,**l**,**n**,**p**,**r**); the means (*n* = 3) labeled with the same letter are not significantly different according to the LSD test at *p* = 0.05 in (**s**–**v**).

The clustered buds derived from the proliferation media were segmented into individual buds and transferred on 1/2 MS medium (Beijing Solarbio Science & Technology Co., Ltd., Beijing, China) supplemented with various concentrations of IBA or NAA for rooting. After culturing for 30 days, the rooting conditions were assessed, and both the number and length of the roots were investigated (Figure 3). The results showed that all the cultured individual buds were able to form roots normally, irrespective of the presence of hormones in the media. In the absence of hormone supplementation in the medium, the buds developed an average of 4.4 roots with a length of 5.87 cm. When 0.1–1.5 mg/L IBA was added to the rooting media, no significant change was observed in the number of roots; however, the root length was significantly reduced. The root numbers for 0.1, 0.5, 1.0, and 1.5 mg/L IBA ranged from 4.6 to 6.7, with no significant differences among them. The root length at 0.1 mg/L IBA was 2.06 cm, significantly lower than that observed at 0.5, 1.0, and 1.5 mg/L IBA (5.00 cm, 4.76 cm, and 4.83 cm, respectively); no significant differences were observed between these three values. When 0.1–1.5 mg/L NAA was added to the rooting media, the root number gradually increased alongside an increase in NAA concentration; however, the root length gradually decreased. Moreover, at an NAA concentration of 0.1 mg/L, the cultured single buds formed 10.1 roots with a length of 3.66 cm. Both of these values were significantly different from those observed in the medium not supplemented with hormones. At an NAA concentration of 1.5 mg/L, the root number increased to 27.7, which was significantly higher than that in the other treatments. Conversely, the root length was only 1.12 cm, which was the shortest among the treatments.

The greatest root number appeared in the medium supplemented with 1.5 mg/L NAA, whereas the longest root length appeared in the medium not supplemented with hormones. To further determine the optimal rooting medium, the rooting seedlings were transplanted into growing media for cultivation and acclimation. After 30 days of cultivation, the results indicated that the growth condition of seedlings derived from the medium not supplemented with hormones was the worst, with a plant height of 18.6 cm and stem diameter of 0.98 mm. The addition of 0.1–1.5 mg/L IBA or NAA to the rooting medium significantly increased these two trait values in the resulting seedlings, with the NAA group values being higher than those of the IBA groups. Interestingly, the plant height of rooting seedlings from the 0.5 mg/L NAA group was 44.84 cm, and the stem diameter was 2.54 mm, both of which represented the highest values across all treatments. Therefore, the optimal rooting medium was established as 1/2 MS supplemented with 0.5 mg/L NAA.

## 3. Discussion

The capacity for differentiation differs greatly among various explant types, which affects the tissue culture effect. Therefore, choosing appropriate explants is crucial for establishing a tissue culture propagation method. Recent studies have shown that explants suitable for tissue culture of *A. donax* include leaves, stem fragments, axillary buds, and immature inflorescences [44,46,51]. The axillary buds (or stem segments containing axillary buds) primarily served as explants for the tissue culture method based on clustered bud proliferation in *A. donax* [44,48,49]. In this study, three distinct types of axillary buds were used as explants to observe their induction effects. The axillary buds with whole cane fragments exhibited a higher induction rate and produced longer buds than those with incomplete cane fragments. This may be attributed to the fact that the complete cane structure enhances the absorption of nutrients by axillary buds, thereby improving sprouting and development. However, axillary buds without cane fragments could not be effectively induced to form clustered buds, and browning was observed. This may be attributed to the direct contact with disinfectants disrupting the structure of the cut surface of the axillary bud, subsequently reducing its vitality. Our results are consistent with a previous report that intact axillary buds on a stem section exhibited superior sprouting compared to detached buds [52]. Therefore, axillary buds with whole cane fragments were identified as the optimal explants for the tissue culture method, as determined by clustered bud proliferation.

Explants may be sourced from wild-, field-, or laboratory-grown plants, often resulting in the presence of some contaminants, including bacteria, fungi, and viruses [53,54]. Therefore, disinfection of explants is crucial for establishing a sterile tissue culture protocol. Currently, the primary disinfectants employed for *A. donax* explants are sodium hypochlorite and mercuric chloride [45,46,51,52]. In this study, various concentrations and treatment times were used, and the disinfection effects were evaluated by investigating contamination and mortality rates. Our results showed that altering the disinfection conditions from 2% sodium hypochlorite for 10 min to 10% sodium hypochlorite for 10 min decreased the contamination rate of the explants from 74.4% to 52.2%, while the mortality rate increased from 0 to 58.9%. These two values were greater than 50%, implying that sodium hypochlorite was unsuitable for disinfecting *A. donax* explants. Utilizing 0.1% mercuric chloride as the disinfectant demonstrated that extending the treatment time from 3 to 5 min decreased the contamination rate of the explants from 35.6% to 17.8% and concurrently increased the mortality rate from 0 to 22.2%. Both these values were relatively low and lower than those of explants treated with 10% sodium hypochlorite for 10 min, thereby indicating that mercuric chloride was more effective for the disinfection of *A. donax* explants than sodium hypochlorite. This finding was consistent with that reported by Lin et al. [51]. Therefore, the explants were subjected to a disinfectant method involving treatment with 0.1% mercuric chloride for 5 min, following a first treatment of 75% alcohol for 30 s.

Nutrients for the explants were provided in the culture medium during the plant tissue culture process. Currently, the *A. donax* tissue culture typically employs MS medium supplemented with various combinations of plant hormones [44,46,49,51]. The various developmental stages of induction, proliferation, and rooting are influenced by adjusting the types and ratios of plant hormones, particularly auxins and cytokinins. Auxins typically function in conjunction with cytokinins to facilitate the differentiation and proliferation of buds and roots. A high auxin to cytokinin ratio facilitates root formation, while a high cytokinin to auxin ratio promotes bud differentiation [55,56,57]. In this study, the MS medium was used as the basic medium. Furthermore, to determine the optimal auxin and cytokinin for inducing clustered buds, we first compared the induction effects of 5.0 mg/L 6-BA in combination with 1.0 mg/L 2,4-D, NAA, and IBA. The results showed that the combination of 5.0 mg/L 6-BA and 1.0 mg/L IBA had significantly higher induction (97.8%) and significantly lower browning (1.1%) rates than those observed for the other two combinations. Notably, the combination of 6-BA and 2,4-D resulted in an induction rate of 3.3% and browning rate of 25.6%. This may be attributed to the report that 2,4-D is primarily used for callus induction and exhibits a specific toxic effect on plant cells [58,59,60]. Subsequently, the induction effects of 1.0 mg/L IBA combined with 5.0 mg/L 6-BA, KT, or TDZ were compared. The results showed that the induction rate for the combination of 5.0 mg/L 6-BA and 1.0 mg/L IBA was also significantly higher than those of the other combinations. In addition, the induction rate of 97.8% and browning rate of 1.1% indicated a relatively good induction effect. Therefore, the effects of other concentrations (or ratios) of 6-BA and IBA were not investigated. The plant hormones in the induction medium were identified as 5.0 mg/L 6-BA + 1.0 mg/L IBA.

In plant tissue culture systems, seedling yield is determined by the proliferation coefficient during the proliferation stage. To achieve a high proliferation coefficient, various media not supplemented with hormones or with differing ratios of 6-BA (0.5–15.0 mg/L) and IBA (1.0 mg/L) were employed to investigate the proliferation effect. The results showed that hormones significantly promoted the proliferation of clustered buds. With an increase in 6-BA, the proliferation coefficient first increased and then decreased, reaching a maximum value of 33.3 at 4.0 mg/L 6-BA, which was significantly higher than those of the other treatments. In addition, the proliferation coefficient was only 4.7 at 15.0 mg/L 6-BA. These results indicate that the proliferation of clustered buds is a complex process, wherein the interaction of multiple hormones, rather than a single hormone, is crucial. Similar results have been reported in several other reports [46,61,62,63]. Therefore, the concentrations of plant hormones in the proliferation medium were determined as 4.0 mg/L 6-BA + 1.0 mg/L IBA.

Tissue culture seedlings require rooting and acclimatization prior to field transplantation [64,65]. Plants promote root growth in response to nutrient deficiencies in their environment [66,67]. Therefore, the basic medium was replaced with 1/2 MS medium at the rooting stage. On this medium, each seedling produced an average of 4.4 roots measuring 5.87 cm in length. Previous studies have demonstrated that auxin is crucial in the rooting stage of plant tissue culture [61,68]. In this study, the addition of 0.1–1.5 mg/L IBA to the medium did not significantly affect root number; however, a significant decrease in root length was observed. Adding 0.1–1.5 mg/L NAA significantly increased the root number laying from 10.1 to 27.7 and significantly decreased the root length laying from 3.66 to 1.12 cm. These results indicate that NAA exhibits good rooting ability in *A. donax* tissue cultures, which is consistent with the findings of previous studies [69,70]. A negative correlation existed between root number and root length; therefore, the rooted seedlings from the various rooting media were acclimated for 30 days to assess the growth conditions. The findings showed that almost all seedlings (>97%) were able to survive at the acclimatization stage, with those derived from the medium supplemented with 0.5 mg/L NAA exhibiting the highest plant height and the largest stem diameter. These findings indicate that both the root number and root length are crucial for the cultivation and acclimatization of *A. donax* seedlings rather than either factor in isolation. This finding is consistent with the fact that a strong root system is characterized by more, longer and thicker roots. Therefore, the concentration of plant hormones in the rooting medium was determined at 0.5 mg/L NAA.

In this study, the disinfectant method resulted in a contamination rate of 17.8%, and a mortality rate of 22.2%; future research should further reduce the contamination and mortality rates of explants to achieve a better disinfectant effect. In addition, the growth condition and yield of obtained seedlings transplanted into an open field were not analyzed, which should also be studied in the future.

## 4. Materials and Methods

### 4.1. Plant Materials

The *A. donax* specimens used in this study were stored at the Henan International Joint Laboratory of Crop Gene Resources and Improvements and subsequently cultivated in the experimental field of the School of Agricultural Sciences, Zhengzhou University. All propagules originated from a single clone to eliminate genetic variation. In March 2022, rhizomes were transplanted into the soil at intervals of approximately 80 cm within 20 m rows, which were separated by 100 cm. Irrigation and weeding were performed in accordance with standard field management practices.

### 4.2. Explant Preparation and Disinfection

Healthy *A. donax* plants of equal sizes were selected for this study. Following leaf removal, the canes were cut into fragments, each containing one node and one axillary bud. Three distinct types of axillary buds were used as explants. First, the fragments measured 0.8–1.2 cm in length above the node and 0.6–1.0 cm long below the node; these explants were designated as axillary buds with whole cane fragments. Second, the fragments were 0.8–1.2 cm long in length above the node and 0.2–0.3 cm in length below the node. The fragments were cut longitudinally, retaining only the sections containing the axillary bud. These explants were designated as axillary buds with incomplete cane fragments. Third, axillary buds were directly cut from the cane fragments. The explants were designated as axillary buds without cane fragments.

The explants were first soaked in a washing liquid solution for approximately 60 min to eliminate surface impurities. Subsequently, they were rinsed under running tap water for 1–2 h. Following initial treatment, the explants were transferred to a benchtop for disinfection. The explants were disinfected with 75% ethanol for 30 s, followed by 2–3 washes with sterile water. After thorough rinsing, the explants were immersed in varying concentrations of sodium hypochlorite or mercury chloride solution for specified durations, followed by 6–8 extensive washes with sterile water. Finally, the explants were dried using sterile filter paper and placed in a clustered bud induction medium. After culturing for 7 days, the contamination and mortality rates of the explants were assessed. Each treatment comprised 30 explants and was conducted in triplicate.

### 4.3. Induction of Clustered Buds

Following disinfection, the explants (axillary buds with whole cane fragments) were placed on Murashige and Skoog (MS) medium supplemented with different combinations of cytokinins and auxins to induce clustered bud formation. First, the explants were placed on the medium supplemented with 5.0 mg/L of 6-benzylaminopurine (6-BA) and 1.0 mg/L of different types of auxins [3-indolebutyric acid (IBA), 1-naphthaleneacetic acid (NAA), and 2,4-dichlorophenoxyacetic acid (2,4-D)]. Following culturing on these induction media for 30 days, the status of the clustered buds was assessed, and both the induction and browning rates were investigated. Each treatment comprised 30 explants and was conducted in triplicate. After identifying the optimal auxin, the explants were subsequently transferred to a medium supplemented with 1.0 mg/L of IBA and 5.0 mg/L of various cytokinin types [6-BA, kinetin (KT), thidiazuron (TDZ)]. Similarly, the status of the clustered buds was assessed, and both the induction and browning rates were analyzed after culturing for 30 days.

### 4.4. Proliferation of Clustered Buds

After cultured for 30 days on the optimal induction medium, healthy clustered buds were divided into single buds containing a small amount of basal tissue. Healthy single buds of equal size were selected as materials for the multiplication culture. Subsequently, the materials were transferred to the MS medium supplemented with no hormones or 1.0 mg/L IBA and different concentrations (0.5, 1.0, 2.0, 3.0, 4.0, 5.0, 7.0, and 15.0 mg/L) of 6-BA for proliferation. Each treatment consisted of 30 single buds and was conducted in triplicate. The proliferation coefficient was measured after culturing for 30 days.

### 4.5. Rooting and Acclimation

Following cultured on the optimal proliferation medium for 30 days, healthy clustered buds were carefully divided into single buds. Seedlings measuring approximately 5.0 cm were selected and transferred to 1/2 MS medium supplemented with different concentrations (0.1, 0.5, 1.0, and 1.5 mg/L) of IBA or NAA. The 1/2 MS medium not supplemented with auxin served as the control. After 30 days of culture, the root number and root length were measured. Each treatment consisted of 30 single buds and was conducted in triplicate.

Subsequently, the rooted seedlings were transplanted into the soil for acclimation. First, the seedlings were trained in the culture bottle for 3 days, during which the bottle cap was loosened, and sterile water was added in appropriate amounts. The trained seedlings were carefully extracted from the bottle, and the culture medium remaining on the roots was removed by gentle washing with running water. The seedlings were subsequently transplanted into a 32-hole cavity tray filled with 75% nutrient-rich soil and 25% vermiculite substrate. The height and stem diameter of the seedlings were measured following a 30-day cultivation period. During this period, the seedlings were watered every 2 days.

### 4.6. Culture Condition

The MS medium served as the basal medium for the induction and proliferation of clustered buds, whereas the 1/2 MS medium was used as the basal medium for the rooting stage. The culture medium contained 30 g/L sucrose (Tianjin Zhiyuan Reagent Co., Ltd., Tianjin, China) and 7.0 g/L agar powder (Beijing Solarbio Science & Technology Co., Ltd., Beijing, China) at a pH of 5.8. Except for having the same composition, the culture media used for the various growth stages contained different types and concentrations of plant hormones. The medium was sterilized in an autoclave at 121 °C for 20 min. The entire culture process was performed in a plant-growth room. The culture temperature was maintained at 25 ± 2 °C, with a 16-8 h day-night cycle and 3000–4000 Lx light intensity.

### 4.7. Statistical Analysis

The induction rate was calculated as the ratio of the number of explants formed clustered buds to the total number of explants in each treatment. The contamination and browning rates were calculated as the ratio of the number of contaminative (or browning) explants to the total number of explants in each treatment. The proliferation coefficient was calculated as the number of buds formed during the proliferation stage divided by the number of cultured buds.

Data were analyzed using Microsoft Excel 2016 (Microsoft Corp., Redmond, WA, USA) and IBM SPSS Statistics (version 20.0; IBM Corp., Armonk, NY, USA). Between-group differences were assessed through analysis of variance and least significant difference (LSD) tests for multiple comparisons. Statistical significance was set at *p* < 0.05.

## 5. Conclusions

We established a tissue culture method for efficiently harvesting *A. donax* seedlings based on clustered bud proliferation. Briefly, the canes were cut into fragments, each containing one node and one axillary bud. These cane fragments served as explants and were first sterilized with 75% alcohol for 30 s and then disinfected with 0.1% mercuric chloride for 5 min. Subsequently, the explants successively underwent induction, proliferation, and rooting stages using MS + 5.0 mg/L 6-BA + 1.0 mg/L IBA, MS + 4.0 mg/L 6-BA + 1.0 mg/L IBA, and 1/2 MS + 0.5 mg/L NAA, respectively. This method induced clustered buds in 97.8% of explants, with each bud achieving a 33.3-fold increase in proliferation on the medium. In addition, all seedlings were able to root, and the rooted seedlings survived and grew to an average of 44.84 cm high and 2.54 mm thick plantlets after a 30-day acclimation period. This study provides a theoretical foundation for the large-scale production of high-quality seedlings. Future studies should assess the viability of scaling up the tissue culturing of *A. donax* and verify its industrial applications.

## Figures and Tables

**Table 1:** Effects of various disinfection methods on explant disinfection.

Disinfection Method	Contamination Rate (%)	Mortality Rate (%)
2% sodium hypochlorite for 10 min	74.4 ± 1.1 ^a^	0 ^d^
5% sodium hypochlorite for 10 min	63.3 ± 1.9 ^b^	17.8 ± 2.9 ^c^
8% sodium hypochlorite for 10 min	61.1 ± 2.9 ^b^	32.2 ± 2.9 ^b^
10% sodium hypochlorite for 10 min	52.2 ± 2.2 ^c^	58.9 ± 2.2 ^a^
0.1% mercury chloride for 3 min	35.6 ± 2.2 ^d^	0 ^d^
0.1% mercury chloride for 5 min	17.8 ± 1.1 ^e^	22.2 ± 1.1 ^c^

Data are presented as mean ± SD (*n* = 3), and the means labeled with the same letter in a column are not significantly different according to the least significant difference (LSD) test at *p* = 0.05.

**Table 2:** Effects of various combinations of cytokinin and auxin on explant induction.

Combinations of Cytokinin and Auxin	Induction Rate (%)	Browning Rate (%)
5.0 mg/L 6-BA + 1.0 mg/L 2,4-D	3.3 ± 1.9 ^e^	25.6 ± 2.2 ^a^
5.0 mg/L 6-BA + 1.0 mg/L NAA	72.2 ± 2.9 ^d^	4.4 ± 1.1 ^b^
5.0 mg/L 6-BA + 1.0 mg/L IBA	97.8 ± 1.1 ^a^	1.1 ± 1.1 ^b^
5.0 mg/L KT + 1.0 mg/L IBA	87.8 ± 1.1 ^b^	5.6 ± 1.1 ^b^
5.0 mg/L TDZ + 1.0 mg/L IBA	81.1 ± 2.2 ^c^	4.4 ± 2.2 ^b^

Data are presented as mean ± SD (*n* = 3), and the means labeled with the same letter in a column are not significantly different according to the LSD test at *p* = 0.05.

## Data Availability

The data are contained within the article.
